# Insights into the regulation of the HOTAIR proximal promoter

**DOI:** 10.1042/BST20260099

**Published:** 2026-09-03

**Authors:** Aritra Gupta, Kartiki V. Desai

**Affiliations:** 1 Biotechnology Research Innovation Council-National Institute of Biomedical Genomics (BRIC-NIBMG), Kalyani, India; 2 PhD Program, Regional Centre for Biotechnology, Faridabad, India; 3 Regional Centre for Biotechnology, Faridabad, India

**Keywords:** Cancer, eukaryotic gene expression, long intervening non-coding RNA

## Abstract

HOTAIR (HOX transcript antisense RNA) is a HOXC-cluster long intervening non-coding RNA (lincRNA) whose cancer relevance is tightly coupled to how its transcription is wired into hormone, hypoxia, inflammatory, and developmental signaling. HOTAIR is known to associate with cancer cell proliferation, motility, tumor invasion, and metastasis. The present mini-review focuses on the regulatory architecture and mechanistic complexity of HOTAIR transcriptional regulation, with emphasis on three organizing principles. First, we consider the impact of promoter choice between a canonical proximal promoter (P1), which supports the 2.2–2.4 kb transcript, and an alternative upstream promoter/TSS (P2), which contributes to context-dependent transcription initiation. Second, we examine the long-distance enhancer–promoter communication between HOTAIR distal enhancer and P1/P2. Third, we summarize the recent epigenetic and epi-transcriptomic mechanisms involved in HOTAIR transcript initiation and elongation. A combination of these events determines isoform-specific transcription to govern cell-type-, context-, and cancer specific modulation of HOTAIR expression that promotes tumor formation and cancer progression. Finally, the review proposes how large-scale RNA datasets, long-read sequencing, and isoform-specific studies can refine our understanding of this versatile lincRNA’s regulation.

## Introduction

HOTAIR (HOX transcript antisense RNA) was discovered within the HOXC locus by Rinn et al., as a ∼2.2 kb long intervening non-coding (linc) RNA that repressed transcription *in trans* across the HOXD locus [1]. This was the first time that HOX-cluster non-coding RNA was linked to chromatin modification-mediated transcriptional silencing of broad regulatory regions encompassing homeobox genes. Gupta et al*.* reported elevated expression in triple-negative breast cancer and demonstrated its ability to enhance the metastatic potential of MDA-MB-231 cells in both *in vitro* and *in vivo* models [2]. Elevated HOTAIR levels now correlate with poor patient survival, increased proliferation, and invasiveness across multiple cancers [3]. While HOTAIR's downstream functions, protein partners, and competitive endogenous RNA networks are reviewed elsewhere [4], the present review focuses on the regulatory transcriptional architecture that determines its isoform, cell type, and context-specific expression.

## Regulatory architecture: canonical proximal promoter, alternate promoter, and a long-range enhancer

**Figure 1:**
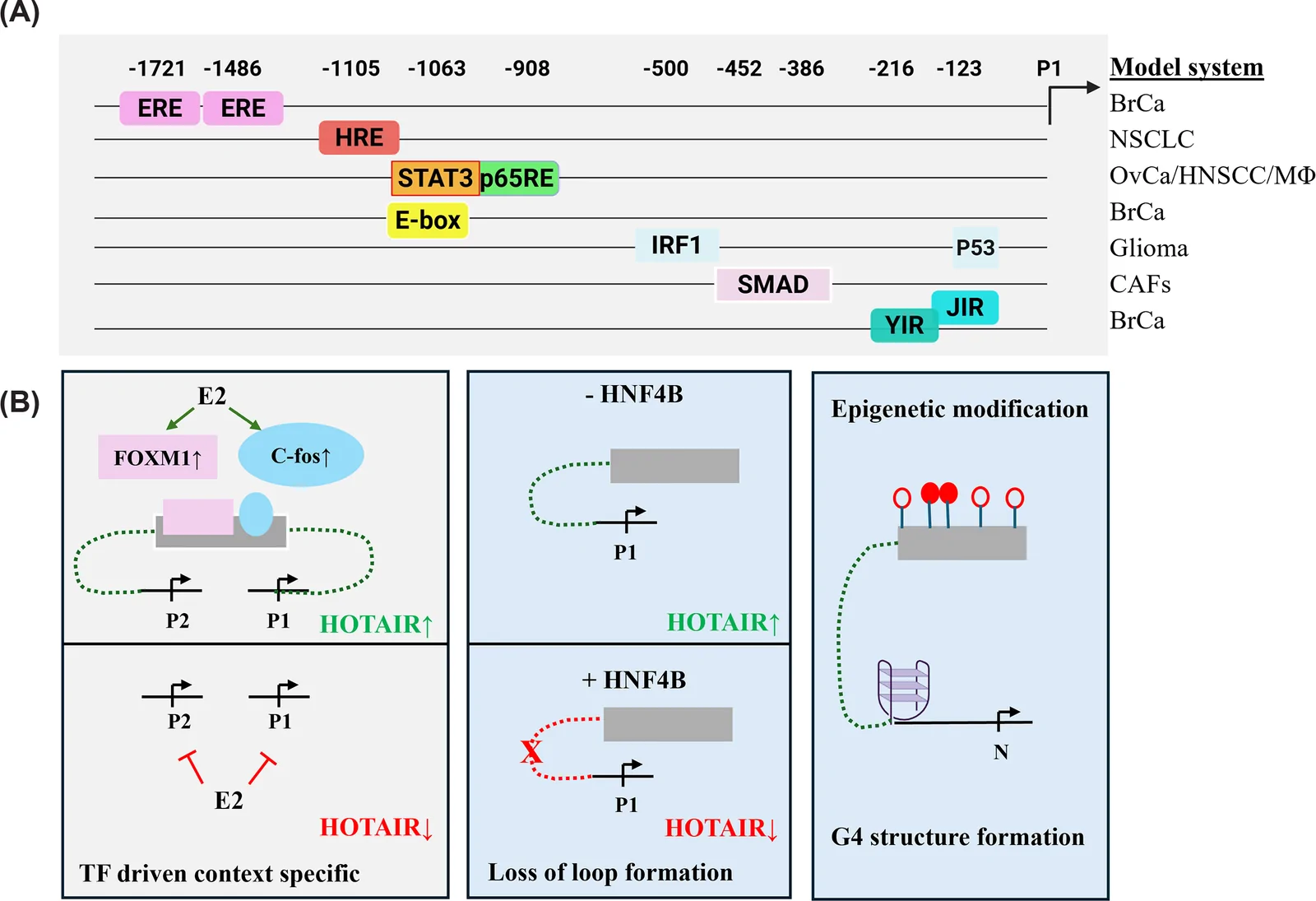
Known regulators of HOTAIR transcription (**A**) TF binding sites are indicated on a solid black line used to represent DNA and the canonical P1 promoter. For simplicity, only a single number is used to show the distance from canonical TSS below the black line. Model systems used are on the right-hand panel. (**B**) HDE-mediated long-range interactions with HOTAIR promoter N, P1, and P2. Gray rectangle represents HDE.

HOTAIR transcription emerges as a mechanistic ‘gateway’ through which cancer cells translate a variety of extracellular cues as diverse as estrogenic signaling, hypoxia, inflammatory stress, and tissue microenvironment (TME) into chromatin regulatory programs. The canonical promoter that supports the widely studied ∼2.2–2.4 kb HOTAIR transcript and includes an upstream alternative/second promoter/TSS (5–8 kb away) and long-range enhancer (150 kb downstream) [5–7]. This distal enhancer (HOTAIR distal enhancer, HDE) forms looping interactions with the canonical (P1) as well as alternate HOTAIR promoter (P2). Such selective enhancer-promoter wiring provides a mechanistic basis for promoter switching, differential responsiveness to transcription factor (TF) binding, and integration of additional signaling inputs that stimulate/suppress factors involved in HOTAIR regulation [7]. This multi-layered architecture explains why HOTAIR expression is highly context-dependent across tumor types and cell states. HOTAIR regulation is complex and intricate, involving both positive and negative feedback loops, multiple TFs, and combinations of regulatory elements. These are described below and summarized in Figure 1. Table 1 indicates the experimental approaches employed and the position of individual TF sites relative to the canonical TSS based on the hg38 version of the reference human genome.

**Table 1:** Summary of validated regulators of HOTAIR transcription

Regulator	Evidence type(s)	Promoter coordinates	Effect	Reference
ERα/ERβ	Promoter centric: ChIP; luciferase; mutagenesis; cofactor recruitment	ERE2 ∼1486; ERE3 ∼−1721	Activation	[6]
ERα	Breast cancer enhancer-centric chromatin maps : HDE, P2/P1 contacts, chromatin marks, lineage TF occupancy, Isoform specific PCR	ER-low/negative or context-dependent HOTAIR expression from P1 or P2	Activation/ Suppression	[7]
FOXM1/c-fos	ChIP peaks in long-range interval; enhancer-promoter looping	Enhancer region (HDE interval)	Activation	[7]
HIF-1α	ChIP; luciferase; EMSA; site substitution	−1153 bp	Activation	[24]
IRF1	ChIP; luciferase	∼500 bp upstream	Repression	[31]
c-MYC/MAX	ChIP; luciferase; E-box mutagenesis; EMSA	E-box at ∼−1053	Activation	[23]
NF-κB/p65 (RelA)	ChIP-qPCR; luciferase; site mutagenesis; stimulus/inhibitor perturbation	Canonical site at ∼−908 bp	Activation	[38]
STAT3	Perturbation (inhibitor)	∼1000 bp	Activation	[39]
SMAD2/3/4	ChIP; luciferase mutational analysis; perturbation	(−386 to −398) and (−440 to −452) bp	Activation	[26]
p53	ChIP; luciferase	Within the basal promoter (−127 and −50) bp	Repression	[32]
BRD4	ChIP; BET inhibitor treatment; rescue	HOTAIR promoter	Activation	[14]
JMJD6	Promoter deletions; ChIP; EMSA; mutagenesis	pHP216 (−216 to +50) minimal; JIR (−123 to −103)	Activation	[17]
YBX1	Luciferase; YIR mutation; ChIP + ChIP–reChIP; EMSA	YIR (−216 to −123); cooperates with JMJD6 at JIR	Activation	[18]
HNF-4α	Luciferase, inhibition	HDE loop removed chromatin topology modification	Repression	[33]

## HOTAIR and breast cancer

### Regulation by estrogen receptor

Typically, estrogen (E_2_) acts by interacting with estrogen receptor (ER), a ligand-activated TF. Primarily, E_2_/ER axis governs cell proliferation and cell physiology of breast cancer cells by transactivating the expression of thousands of genes via binding regulatory DNA elements in a sequence-specific manner. These sequences are termed as estrogen-responsive elements (EREs). EREs have been thoroughly characterized by cellular and molecular biology approaches and by more advanced chromatin immunoprecipitation techniques. Literature suggests that E_2_ regulates HOTAIR transcription both positively and negatively. A primary mechanistic map of the HOTAIR P1 promoter showed the presence of four promoter-proximal EREs. Two functional EREs (ERE2 and ERE3) mapping to approximately −1486 bp and approximately −1721 bp upstream of the HOTAIR TSS could enhance luciferase activity in the presence of the hormone. ERα/ERβ occupancy of these sites was supported by ChIP assays, and ERE mutagenesis diminished promoter luciferase activity [6]. ER recruitment was coupled with coactivator binding and chromatin-modifier engagement via p300/CBP and MLL family proteins, resulting in the deposition of permissive chromatin marks such as increased H3K27 acetylation and H3K4me3 localization. Further, estrogenic endocrine disrupting chemicals (EDCs) and synthetic estrogens that can bind ER at extremely low concentrations also resulted in HOTAIR dysregulation via the same EREs [8]. These data connect EDC exposure to lincRNA oncogenesis and provide additional confirmation for a positive association between ER and HOTAIR expression.

In contrast, other studies report a negative association between estrogen treatment of MCF7 cells and HOTAIR expression [7,9]. Indeed, observations from our lab also suggest that higher HOTAIR RNA levels were associated with decreased ER expression (see section below). Milevskiy et al. discovered the HDE locus and the alternate promoter P2 in MCF7 breast cancer cells using available promoter-associated chromatin signatures in breast cancer. They showed that FOXM1 and c-fos physically interact with HDE locus and induced both the short (P1-driven) and long (P2-driven) HOTAIR transcripts. Surprisingly, while E_2_ enhanced the expression of FOXM1 and c-fos, it suppressed the expression of both HOTAIR RNA isoforms. High HOTAIR expression in tamoxifen-resistant (TAMR cells) further supported the negative correlation observed for ER and HOTAIR [9]. However, HOTAIR RNA binding in turn stabilized ER protein, augmenting ER-mediated transactivation. This established a positive feedback loop supportive of endocrine therapy resistance and cancer recurrence [10]. These results are not necessarily contradictory and could reflect different regulatory layers: acute estrogen-responsive activation of P1 in ER-positive cells versus enhancer-driven or P2-biased HOTAIR expression in cell states where ER is low, absent, or functionally bypassed. This model can be tested by combining ER perturbation with isoform-specific RT-qPCR, 5′ RACE/CAGE, long-read RNA-seq, HDE/P1/P2 CRISPRi and 3C/HiChIP analysis across ER-positive, ER-negative and endocrine-resistant models. Such divergent ER- regulatory models may also arise due to methodological differences in experimental design, duration of estrogen treatment, and model systems used (acute short-term exposure in cells versus long-term endocrine therapy treatment). Further, lack of isoform-specific PCR analysis, under-explored differential chromatin states, and differences in laboratory clones of cell lines.

In ER-negative cells, such as triple-negative (TNBC) cells, estrogen-driven non-genomic actions augmented HOTAIR expression via GPER-mediated miR148a expression [11]. This implies that indirect mechanisms that have not yet been linked to the exact promoter-driven events exist, revealing the complexity of the HOTAIR dysregulation.

### The JMJD6, MYC, and YBX1 loop

In MCF7 and MDA MB 231 breast cancer cells, we encountered HOTAIR as a gene positively regulated by JMJD6. Their co-expression in breast tumors was associated with worst survival outcomes [12]. JMJD6 is an arginine demethylase and lysyl hydroxylase that modifies several proteins including ER, JMJD6, p53, and histones. The latter ability makes it an epigenetic regulator of transcription. It enhances transcription by directly binding and licensing several factors such as ER and BRD4 and mediates the release of paused RNA polII sites to enable transcript elongation [13]. Of these TFs, treatment of glioblastoma cells with anti-proliferative inhibitors against BRD4 protein like JQ1(BET inhibitors) reduced HOTAIR levels [14] and both JMJD6 and BRD4 were implicated in promoting radioresistance in hepatocellular carcinoma stem cells via their ability to bind and regulate HOTAIR [15].

In MCF7 cells, neither loss of ER expression in JMJD6-overexpressing cells nor BRD4 presence explained JMJD6-mediated modulation of HOTAIR [16]. The characterization of the P1 HOTAIR promoter in these cells identified a JMJD6-interaction element (JIR) at (−123 to −103 bp). Enzymatic activity of JMJD6 was required for transcriptional induction since mutant JMJD6 bound JIR but could not induce promoter activity, indicating the existence of a two-step mechanism including binding plus catalysis [17]. Since JMJD6 lacks any known DNA binding properties, in our follow up study, we showed that YBX1 occupied an adjacent region (YIR, within −216 to −123), increased luciferase activity of proximal promoter constructs, and is required for stable retardation complex formation using both YIR and JIR probes. ChIP- and ChIP–reChIP-supported co-occupancy of YBX1 and JMJD6 on the HOTAIR promoter, implying YBX1 acts as a recruiter/anchoring factor for JMJD6 [18]. However, our study did not explore P2 activity nor the contribution of the HDE locus. However, our RNA-seq data indicated a positive correlation between JMJD6 and FOXM1 levels (but not c-fos), suggesting additional studies using HDE are necessary.

**Figure 2:**
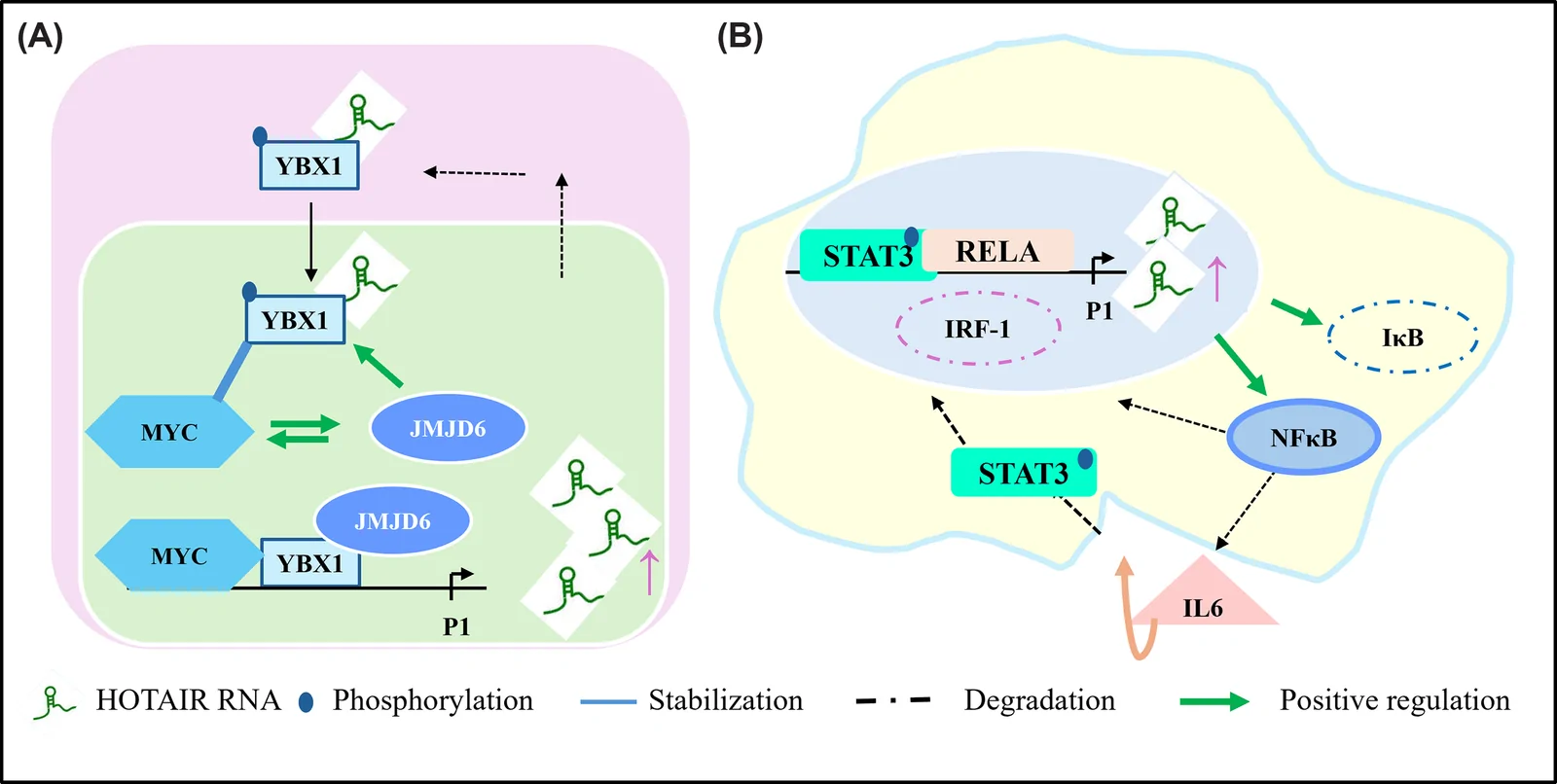
Proposed positive feedback loops operating to reinforce HOTAIR-driven oncogenic functions (**A**) In cancer cells and (**B**) In both cancer cells and macrophages.

Another key regulator of HOTAIR promoter is c-MYC, and its overexpression in breast cancer cells enhanced HOTAIR expression [19]. Interestingly, MYC is known to enhance JMJD6 levels by direct interaction with the JMJD6 proximal promoter [20], and JMJD6 positively regulates MYC [21]. YBX1, on the other hand, is known to interact with MYC mRNA and stabilize it thereby enhancing MYC functionality in cancer cells [22]. Together, these data indicate a positive feedback loop of HOTAIR-MYC-JMJD6-YBX1 regulation that culminates in cancer progression (Figure 2A). In gall bladder cancer, c-MYC activates the HOTAIR P1 promoter, and this was paired with suppression of miR-130a, illustrating how promoter activation can be coupled to post-transcriptional pathways [23]. Since TF biology is highly context- and enhancer-dependent, generalization across cancers will be best supported when overlapping TF ChIP peaks across cancers within the HOTAIR regulatory region are experimentally demonstrated or when CRISPRi/CRISPR-editing confirms necessity of TF specific sites for HOTAIR regulation.

## HOTAIR expression in other cancers

HOTAIR canonical promoter activity is strongly correlated with many cancers and their cell-autonomous and non-cell-autonomous interactions. Cancer cells acquire several hallmark properties by modulating their responsiveness by physical contact with cells of the tumor microenvironment and respond to extracellular matrix and soluble factors secreted by immune cells, fibroblasts, and endothelial cells. Cancers adapt to hypoxic environments and deposit excessive type I collagen. Under hypoxic environments, NSCLC cells expressed HIF-1α to induce HOTAIR transcription, helping them adapt to low oxygen availability in the TME [24]. Similarly, increased Type I collagen deposition and collagen-up-regulated HOTAIR RNA, though details of TFs/promoter sites used for this regulation have not been investigated [25]. Cancer-associated fibroblasts promote cancer growth by releasing tumor-promoting factors such as TGFβ, which in turn can up-regulate HOTAIR expression by increasing SMAD2/3/4 binding at the HOTAIR promoter [26]. Similarly, components of inflammatory response or cellular immune signaling are often activated within the cancer cells following their exposure to TME. Here, HOTAIR regulation along with its downstream functional impact was shown to converge on the NF-κB pathway. Regulatory binding sites for this factor, STAT3 and IRF-1, have been identified within 1 kb of the canonical TSS in ovarian cancer, gliomas, and HNSCC, respectively [27–29]. Signals as diverse as DNA damage response and/or cytokine signaling via IL6/STAT3 were responsible for changes in HOTAIR transcription. Often, induced HOTAIR levels maintained heightened signaling by degrading the NF-κB inhibitor, IκB, to establish a positive regulatory loop. An increase in NF-κB in macrophages following bacterial liposaccharide stimulation led to a similar increase in HOTAIR RNA and HOTAIR-dependent IL6 release and iNOS induction [30]. The released IL-6 was shown to reinforce the activation of NF-κB, establishing a positive feed-forward loop (Figure 2B). The source of these cytokines may be tumor cells, stromal cells, infiltrating immune cells, or a combination of these depending on tumor context; however, direct immune-cell-source attribution remains insufficiently resolved in most published studies.

On the other hand, few repressors of HOTAIR expression have been studied. Osteopontin-driven signaling augments IRF1, and its binding to HOTAIR promoter decreases transcriptional activity, identifying a transcriptional repressor [31]. In our studies, we inadvertently deleted the putative IRF-1 site and found that HOTAIR promoter constructs were hyperactivated, and their promoter activity was uncoupled from the JMJD6 axis [17]. Further, IRF-1 expression itself was highly suppressed by JMJD6 (personal observations), suggesting that JMJD6 may promote HOTAIR transcription by repressing a suppressor. Other reports of HOTAIR suppression in NSCLC models show direct interaction of p53 with HOTAIR canonical promoter and increased HOTAIR-mediated deposition of H3K27me3 marks at the p53 promoter. This study discovered yet another mechanism by linking promoter output to downstream chromatin feedback via reciprocal silencing of a tumor suppressor [32]. Another factor known to directly repress HOTAIR expression in hepatocytes is the nuclear orphan receptor hepatocellular nuclear factor 4-alpha (HNF4-α) [33]. During epithelial *trans*-differentiation, HNF4-α disallows chromatin loop formation between the HOTAIR promoter and the HDE region, ultimately repressing HOTAIR to maintain the epithelial state of the hepatocytes. Overall, the net effect of these events may allow immune escape, promote stemness and anticancer drug resistance, and/or cellular senescence in cancer cells.

## DNA methylation as a regulatory switch

Apart from TF usage, promoter accessibility, and altered histone landscapes, gene regulation is also controlled by direct biochemical modification of the regulatory sites by DNA methylation. The position of CpG methylation often indicates if the associated gene is induced/suppressed. For example, most promoter CpG island hypermethylation leads to gene suppression, whereas gene-body methylations have context-specific outcomes. A breast cancer cohort study quantified methylation surrounding the HOTAIR gene and found a downstream intergenic CpG island (between HOTAIR and HOXC12, designated as DS-CGI) having a positive correlation with HOTAIR expression. This is consistent with a *cis*-regulatory model in which methylation state influences transcriptional read-through/termination and/or locus insulation [34]. However, more recent work challenged DS-CGI using perturbation experiments of transcriptional kinase activity and chromatin readouts in cell lines. The data identified an intragenic methylation at an exon 4-overlapping CpG island as the functional driver and the key causal element (Ex-CGI). Ex-CGI methylation promoted transcription elongation and full-length HOTAIR output. This study further suggested that MLL1-mediated deposition of the H3K4me3 mark enhanced Ex-CGI DNA methylation, and CDK7-CDK9 activity sustained RNAPII Ser2 phosphorylation thereby creating a positive feedback loop for efficient transcriptional elongation [35]. Therefore, DS-CGI methylation may reflect a *cis*-regulatory state that drives HOTAIR transcript initiation, whereas Ex-CGI methylation may act more directly at the elongation step. Choice of either/or both mechanisms may govern context-specific expression of HOTAIR. A decisive test would jointly quantify DS-CGI methylation, Ex-CGI methylation, P1/P2 usage, nascent transcription, and full-length isoform production in the same cellular models before and after targeted methylation editing or CDK7/CDK9 inhibition.

While we have documented several regulators of HOTAIR, a recent report found an alternate HOTAIR-N transcript that began as an antisense to HOXC11 exon and resulted in an alternate HOTAIR RNA. This RNA promoted cell proliferation, death, and survival networks in cancer cells [36]. The promoter of this transcript contained CpG islands harboring G-quadruplex structure that was associated with active transcription marks, triangulating methylation, histone modification landscapes, and DNA structure as elements critical for promoter selection, usage, and regulation. In another study, long-read capture sequencing in human adipose stem cells revealed that HOTAIR existed as a pool of isoforms with multiple start sites and that differentiation triggers a shift in TSS usage. The study explicitly concluded that distinct promoter usage regulates isoform balance and that many isoforms can truncate or alter canonical PRC2/LSD1-binding regions [37]. These recent studies indicate that our mechanistic studies on HOTAIR promoter regulation are far from complete.

## Limitations

The current understanding of HOTAIR regulation is limited to promoter-proximal regions encompassing a small upstream window of about 2 kb of the canonical P1 transcript. Though one HDE element has been characterized for E_2_-mediated HOTAIR transcript, missing are data and analysis of long/distal looping enhancers and their impact on HOTAIR transcription across various cancers, models, and cellular states to improve the generality of findings. Promoter-reporter constructs often test isolated promoter fragments and therefore cannot capture long-range interactions, looping, chromatin insulation, or promoter competition and promoter choice. Bulk qPCR or RNA-seq measurements frequently quantify total HOTAIR without resolving which of the 14 reported transcripts the signal is derived from (https://asia.ensembl.org/). These data measured by non-isoform-specific primers could conflate promoter activation with promoter switching, and isoform-resolved readouts are needed to map TF regulation to the canonical ∼2.2–2.4 kb transcript versus alternative promoters that have been described for the same gene. Individual TF-promoter studies are not always supported by chromatin modification data nor the status of epigenetic or regulatory DNA structural data.

## Perspective and future directions

The present review summarizes the involvement of TFs, hormones, immune regulation, and signaling events, as well as epigenetic regulation of the HOTAIR, and highlights the multiple positive feed forward regulatory loops involved in aggressive cancer behavior. HOTAIR depletion can be achieved by targeting the RNA itself using siRNA, antisense oligonucleotides, GapmeRs, or RNA-targeted delivery systems. While direct HOTAIR depletion remains conceptually attractive, it faces delivery, specificity, and tumor-heterogeneity barriers. Targeting HOTAIR’s regulatory dependencies, such as using BET/BRD4 inhibitors to decrease HOTIAR synthesis or CDK7/CDK9 inhibitors to prevent transcription-elongation as implicated by the Ex-CGI-dependent mechanisms. EZH2/PRC2 inhibitors may not suppress HOTAIR expression itself but could blunt downstream HOTAIR-associated chromatin repression. These approaches should not be framed as universal HOTAIR therapies; rather, they are most plausible in tumors where HOTAIR expression is high and mechanistically coupled to the targeted regulatory module (Table 2).

**Table 2:** Targetable HOTAIR regulatory mechanisms

Targetable node	Rationale	Potential agents/approach
HOTAIR RNA	Direct depletion of oncogenic lincRNA	siRNA, ASO, GapmeR, RNA-targeted delivery
BRD4/BET	BRD4 occupancy supports HOTAIR transcription in selected models	JQ1 and clinical BET inhibitors
CDK7/CDK9/RNAPII Ser2	Elongation control of full-length HOTAIR through Ex-CGI module	CDK7/CDK9 inhibitors
YBX1 protein	MYC-JMJD6-YBX1-HOTAIR loop	YBX1 inhibitors, since MYC is not tuneable JMJD6′s involvement in basic transcription may result in side effects
EZH2/PRC2	Downstream HOTAIR chromatin effector complex	EZH2 inhibitors
m6A machinery	May regulate HOTAIR-protein interactions and chromatin function	METTL3/METTL14/WTAP or YTHDC1 axis

In the future, more details of HOTAIR regulation will be revealed by refinement of *in silico* meta-analysis of large multigenomic methylome, transcriptome datasets obtained from TCGA (https://www.cancer.gov/ccg/research/genome-sequencing/tcga), TF perturbation experiments, Hi-C and histone modification datasets (ENCODE: https://www.encodeproject.org), and RNA pull down linked proteomics. In a small way, we were successful in predicting YBX1 as a potential DNA-binding partner of JMJD6 using such approaches, demonstrating the usefulness of such databases. In addition, novel strategies using long-read sequencing of DNA to detect methylation status in the regulatory regions and long-read sequencing of RNA to determine HOTAIR isoforms will be critical to integrate regulation and expression data. Discovery of these mechanisms will be incomplete without experimental validation using technologies such as CRISPRi across multiple cancer models, factor and binding site mutagenesis, and promoter reporter assays based on long-range interactions.

## Summary

- The regulation of HOTAIR expression suffers methodological and structural bottlenecks due to inclusion of narrow genomic window (∼2 kb upstream) of P1 promoter, lack of analysis of long-range looping elements and conflated non-isoform specific readouts.
- Future research on HOTAIR regulation should aim to overcome traditional analytical limits through advanced genomic modeling and targeted validation by utilizing the power of meta-analysis of integrated datasets across multiple cancers and newer verification technologies.
- HOTAIR expression is driven and reinforced by multiple self-sustaining positive feedback loops that accelerate tumor progression. A deeper analysis could reveal additional loops for the development of novel therapeutic strategies against the RNA itself and its functional partners.

## Open Access

Open access for this article is provided via our Subscribe to Open programme, made possible through the support of our subscribing institutions. Learn more here: https://portlandpress.com/pages/open_access_options_and_prices#conditional.

## Abbreviations

E_2_: estrogen
EDCs: endocrine disrupting chemicals
ER: estrogen receptor
EREs: estrogen responsive elements
HNF4-α: hepatocellular nuclear factor 4-alpha
HDE: HOTAIR distal enhancer
HOTAIR: HOX transcript antisense RNA
JIR: JMJD6-interaction region
lincRNA: long intervening non-coding RNA
TME: tissue microenvironment
TF: transcription factor
